# A Prospective Observational Study Assessing the Relationship Between Solitary Thyroid Nodule Size and Incidence of Malignancy

**DOI:** 10.7759/cureus.11422

**Published:** 2020-11-10

**Authors:** Sherif Monib, Nicholas Farkas, Mohamed I Abdelaziz

**Affiliations:** 1 Breast Surgery, West Hertfordshire Hospitals NHS Trust, St. Albans and Watford General Hospitals, London, GBR; 2 Surgery, West Hertfordshire Hospitals NHS Trust, St. Albans City Hospital, St. Albans, GBR; 3 Surgery, Fayoum University Hospital, Fayoum, EGY

**Keywords:** thyroid, solitary thyroid nodule, ultrasound scan, thyroid cancer

## Abstract

Background

Solitary thyroid nodule (STN) is a well-documented entity. Autopsy data indicate a 50% prevalence of thyroid nodules >10 mm in patients without clinical evidence of thyroid disease. Prevalence of palpable nodules is 4-7%. Solitary thyroid nodules are often asymptomatic and found incidentally. Fine needle aspiration cytology is recommended to determine the nature of the thyroid nodule. 5-10% of the thyroid nodules are found to be malignant following thyroidectomy.

Objective

Our study aims to explore the relationship between solitary thyroid nodule size and malignancy.

Methods

A prospective, observational analysis looking at preoperative thyroid ultrasound scan findings and post-operative histology for a total of 100 female patients referred to our unit within a university hospital from November 2016 to April 2019. Statistical analysis including One-Way ANOVA was performed where appropriate.

Results

Total number of patients was 100 female patients divided according to the size of the nodule into three groups with the correlation between the size of the nodule and the incidence of malignancy.

Group A: Patients with a STN <20 mm; eight patients; post-operative histology = all benign.

Group B: Patients with a STN measuring 20-40 mm; 80 patients: 68 patients were benign, and 12 patients (12%) were malignant (incidence of malignancy in the group is 15%).

Group C: Patients with a STN >40 mm; 12 patients: eight patients were benign, four patients were malignant, (incidence of malignancy = 33%).

Correlation between the size of the nodule and the incidence of malignancy:

Group A: 0/8 malignancy; Group B: 12/80 patients were malignant; Group C: 4/12 malignant.

Conclusion

Our results suggest that the size of a solitary thyroid nodule cannot be reliably used for at predicting malignancy and should not be influencing patient’s management.

## Introduction

Solitary thyroid nodule is a common entity with autopsy data indicating a prevalence of 50% in patients with nodules larger than 1 cm without clinical evidence of thyroid disease. The prevalence of palpable nodules is 4-7% [1, 2].

Thyroid nodules warrant removal when they are large enough to be symptomatic, or if there is a concern for malignancy. The majority of nodules are asymptomatic. With 5-10% of nodules being malignant, the decision to operate is made on therapeutic or diagnostic grounds [3, 4].

Most patients present with a large palpable thyroid nodule. However, some solitary thyroid nodules are incidentally found on imaging studies performed for other reasons [5].

Barroeta et al. found that a single dominant or solitary nodule is more likely to represent carcinoma than a single nodule within a multi-nodular gland. The incidence of malignancy increased from 2.7 to 30% and from 1.4 to 10%, respectively [6].

Important elements in a patient's history increase the likelihood of malignancy, these include prior head and neck irradiation, rapid nodule growth, dysphagia, dysphonia, male gender, presentation at extremes of age (<20 years or >70 years) and family history of medullary thyroid carcinoma or multiple endocrine neoplasia [7,8].

Aim of our study

This study aims to determine the relationship between size and the incidence of malignancy in patients with a solitary thyroid nodule.

## Materials and methods

We conducted a prospective, observational analysis looking at preoperative thyroid ultrasound scan findings and post-operative histology for a total of 100 female patients referred to our unit within a university hospital from November 2016 to April 2019.

The study was approved by the local medical ethics committee. Inclusion criteria: female patients only (to eliminate sex-related bias) who were found to have a solitary thyroid nodule on neck ultrasound scan. All selected patients were euthyroid and fit for general anaesthesia. Exclusion criteria: male patients, patients with diffusely enlarged thyroid gland, patients not fit for general anaesthesia, recurrent cases and patients with thyrotoxicosis.

All patients were euthyroid on preoperative blood tests and underwent a preoperative neck ultrasound scan and an ultrasound-guided fine-needle aspiration cytology (FNAC).

Statistical analysis

Data were collected and coded to facilitate data manipulation and double entered into Microsoft Access and data analysis was performed using Statistical Package of Social Science (SPSS) software version 18 (IBM Corp., Armonk, NY) in windows 7.

## Results

Total number of patients included in our analysis was 100 patients; their age range was 20-55 years with mean ± SD 33 ± 9.5 years, 56 patients (56%) were younger than 30 years, 28 patients (28%) were 30-40 years, 12 patients (12%) were 40-50 years and four patients (4%) were older than 50 (Table 1).

**Table 1:** Distribution of studied sample according to patient’s age

Age	No.	%
<30 y	56	56%
30-40 y	28	28%
40-50 y	12	12%
>50 y	4	4%
Total	100	100%
Min.-Max.	20-55 ys	
Mean ± SD	33.00 ± 9.5 ys	

Patients were divided according to the maximum diameter size of the solitary thyroid nodule on ultrasound scan. Similar categories were used for both pre-operative and post-operative size: group (A): less than 20 mm, group (B): 20-40 mm, and group (C) greater than 40 mm. Pre-operative ultrasound and post-operative histopathology reports demonstrated minimal difference ±1-2 mm between the two groups (Table 2).

**Table 2:** Distribution of the studied sample according to the nodule size in preoperative ultrasound (US) compared to nodule size in postoperative histopathology reports.

Nodule size in each group	Size by US	Size in post-operative pathology
Group (A): size < 2 cm	8 (8%)	8 (8%)
Group (B): size 2-4 cm	80 (80%)	80 (80%)
Group (C): size >4 cm	12 (12%)	12 (12%)
Total	100 (100%)	100 (100%)
Min.-Max.	1.5 cm-5 cm	
Mean ± S.D.	3.5 cm ± 1.5 cm	

Pre-operative FNAC revealed that 20 patients (20%) had colloid adenoma, 48 patients (48%) had a follicular lesion, 28 patients (28%) had papillary adenoma, four patients (4%) had papillary thyroid carcinoma (Table 3).

**Table 3:** Distribution of studied sample according to pre-operative FNAC results. FNAC: Fine-needle aspiration cytology

Pre-operative FNAC	Number	%
Colloid adenoma	20	20%
Follicular lesion	48	48%
Papillary adenoma	28	28%
Papillary carcinoma	4	4%
Total	100	100%

A total of 80 patients (80%) had hemi-thyroidectomy of the affected lobe. Exploration of the contralateral lobe was carried out for all patients. Fine nodularity was found in 20 patients (20%). Total thyroidectomy with lymph node dissection was carried out for the four patients with carcinoma.

Post-operative histopathology results were divided into two groups:

Benign: 84 patients (84%) - 20 colloid adenoma, 36 follicular adenoma, 20 patients (20%) were papillary adenoma and eight patients (8%) were papillary adenoma with cystic degeneration; Malignant: 16 patients (16%) - 12 follicular carcinoma and four papillary carcinomas (Table 4).

**Table 4:** Distribution of studied sample according to patient’s post-operative histopathology.

Post-operative histopathology	No	%
Colloid adenoma	20	20%
Follicular adenoma	36	36%
Papillary adenoma	20	20%
Papillary adenoma & cystic degeneration	8	8%
Follicular carcinoma	12	12%
Papillary carcinoma	4	4%
Total	100	100%

Based on histological size of the solitary thyroid nodule, patients were divided into three groups; Group A: (thyroid nodule < 20 mm) included eight patients (8%), all had follicular adenoma. Group B: (thyroid nodule 20-40 mm) 80 patients (80%), of which 68 patients were benign, 12 patients (12%) were malignant (incidence of malignancy in the group is 15%). Breakdown of histology: 16 patients had a colloid adenoma, 28 patients with papillary adenoma, 24 patients with follicular adenoma, eight patients with follicular carcinoma and four patients had papillary carcinoma. Group C: (thyroid nodule > 40 mm) included 12 patients (12%), eight patients were benign (four patients had a follicular adenoma, four patients colloid adenoma), four patients were malignant (follicular carcinoma). The incidence of malignancy in this group was 33%. Nodule size ranged between 1.5-50 mm (mean ± SD is 3.5 ± 1.5 cm) (Table 4).

Correlation between the size of the nodule and the incidence of malignancy:

Group A: 0/8 malignancy. Group B: 12/80 patients were malignant. Group C: 4/12 malignant (Table 5).

**Table 5:** Distribution of studied sample according to patient’s post-operative histopathology in correlation with the incidence of malignancy. * The percentage to the total number in the study (n = 100)

Groups of nodule size	Benign No. (%)	Malignant No. (%)
Group A: <20 mm	8 patients (8%)*	0 (0%)*
Group B: 20-40 mm	68 patients (68%)*	12 patients (12%)*
Group C: >40 mm	8 patients (8%)*	4 patients (4%)*

## Discussion

Thyroid nodules are observed in 8% of the adult population and seen more frequently in women than men. A solitary thyroid nodule is defined clinically as a localized thyroid enlargement with an apparently normal remaining gland. Such nodules carry a 5-15% prevalence of malignancy. Thyroid malignancies account for approximately 1% of all malignant neoplasms and are the most common endocrine neoplasia. With the use of ultrasound, up to 10 times more nodules are thought to be detected. They are found in 4%-8% of adults by palpation and in 13%-67% when ultrasound detection is utilized. In autopsy studies, they have a prevalence of approximately 50% [9-11].

Tai Jun et al. concluded that male gender, microcalcification and lymphadenopathy are independent risk factors for predicting malignancy in patients with STN; patients with more than two of those risk factors should be subjected to further examination or thyroidectomy [9].

Some studies suggested that the size of a solitary thyroid nodule can be considered as an independent predictor for risk of malignancy, especially in the presence of underlying risk factor. McCoy et al. concluded that due to high false-negative rate for preoperative benign cytology, thyroid nodules greater than or equal to 4 cm should be considered for diagnostic lobectomy regardless of fine-needle aspiration biopsy (FNAB) results [12,13].

In our study, patient age ranged between 20-55 years with mean ± SD 33 ± 9.5 years demonstrating a younger age on average when compared with other studies. Godazandeh et al. found the mean ages of the patients to be 41.56 ± 13.24 years (between 13 and 86 years old) [14].

Pre-operative FNAC results contrasted with the final post-operative histology results as it showed that 48% had follicular lesions, and only picked four cases of papillary carcinoma.

Fine-needle aspiration cytology of thyroid nodule was introduced over 50 years ago, and quickly gained popularity to be an important part of thyroid nodule assessment, yet 20% of samples were reported to be indeterminate [15,16]. Musani et al. found in their study that the sensitivity of FNAC of thyroid nodules was 61.53% and specificity was 98.9% [17]. Shere et al. divided the patients according to the result of pre-operative FNAC into four groups: inadequate, benign, malignant, and suspicious. Results demonstrated that FNAC cannot distinguish between follicular adenoma and follicular carcinoma [18].

Intra-operative exploration of the contralateral thyroid lobe in our patients demonstrated that 80% of the contralateral lobes were free from nodularity while 20 patients (20%) showed fine nodularity. Twelve patients underwent total thyroidectomy. McCoy et al. found that in 70% of their patients the contralateral lobe was free from nodularity, in 30% of patients the contralateral lobe demonstrated fine nodularity, whilst 5% of patients underwent total thyroidectomy [13]. Both our results and those of other studies highlight the importance of intra-operative exploration of the contralateral thyroid lobe in order to identify any nodularity not identified on pre-operative neck ultrasound scan.

When looking at the correlation between the size of the nodule and the incidence of malignancy, we found that smaller nodules appear linked to benign findings, whilst larger nodules were associated with a greater incidence of malignancy. Kamran et al. reported that increasing thyroid nodule size had an influence on cancer risk in a nonlinear fashion with no increase in risk beyond the 20 mm threshold [5]. McCoy et al. reported a higher prevalence of thyroid carcinoma in nodule sizes ≥40 mm [13]. Berker et al. also described no significant difference between 10 mm and 40 mm nodules in thyroid malignancy risk [19]. Conversely, Rausei et al. and McHenry et al. reported a higher prevalence of thyroid carcinoma in smaller nodules [20,21].

Three studies demonstrated a higher risk of malignancy in nodules ≥40 mm compared to <40 mm: Bestepe et al. reported a risk of malignancy 24% in nodules ≥40 mm versus 12% in nodules <40 mm in 571 patients [22], Kuru et al. [23] reported a risk of 58.2% in nodules ≥4 cm versus 37.3% in nodules <40 mm in 159 patients and Kamran et al. [5] reported a risk of 15% in nodules ≥40 mm versus 12.3% in nodules <40 mm in 4955 patients.

In our study, we observed that the incidence of malignancy in nodules <40 mm was 15% versus 33% in nodules >40 mm. The risk of malignancy in each group does not appear to increase in a linear manner, and One-Way ANOVA showed no statistically significant difference between malignant and benign nodules in size. Therefore nodule size cannot be considered as a dependent risk factor of malignancy in STN.

There is no international consensus linking an increased risk of malignancy with increased size of thyroid nodules. Therefore thyroid nodule evaluation should aim to accurately assess the malignancy risk via methods that are accurate, precise, yet also safe and cost-effective [24].

Ozel et al. concluded that thyroid nodules can be characterized effectively by using ultrasound scan yet ultrasound features used in this distinction vary in relation to nodule size [25]. Zhao et al. concluded that nodule size estimated by ultrasound (US) shows relatively good correlation with final pathologic size. However, thyroid nodules should undergo FNA regardless of size [26].

While Hong et al. found that the impact of nodule size on the malignancy risk differed according to the US pattern. A large nodule size ≥30 mm showed a higher malignancy risk than smaller nodules in intermediate- and low-suspicion nodules [27]. Zhao et al. concluded that thyroid nodule size is inversely related to malignancy risk, as larger nodules have lower malignancy rates [26]. Our findings support the results of other studies highlighting that there is still uncertainty in relation to the impact of the size of a solitary thyroid nodule and the risk of malignancy.

## Conclusions

Management of solitary thyroid nodules is based on clinical assessment, ultrasound scan findings and preoperative fine-needle aspiration cytology findings. Our results suggest that increasing size of a solitary thyroid nodule may be a predictor for malignancy.

Nevertheless, size of STN cannot be reliably used for predicting malignancy; we advocate thorough pre-operative workup to ensure potential malignancies are not missed. We recommend a multicentric study including a large number of patients to ascertain the relation between size and incidence of malignancy in a solitary thyroid nodule.
